# Descending Control of Nociceptive Processing in Knee Osteoarthritis Is Associated With Intracortical Disinhibition: An Exploratory Study

**DOI:** 10.1097/MD.0000000000003353

**Published:** 2016-04-29

**Authors:** Maria da Graca L. Tarragó, Alícia Deitos, Aline Patrícia Brietzke, Rafael Vercelino, Iraci L. S. Torres, Felipe Fregni, Wolnei Caumo

**Affiliations:** From the Post-Graduate Program in Medical Sciences (UFRGS) (MDGLT, AD, APB, ILST, WC), School of Medicine, Universidade Federal do Rio Grande do Sul; Department of Surgery (WC), School of Medicine at UFRGS; Department of Pharmacology of Instituto de Ciencias Basicas da Saude at UFRGS (ILST); Post-Graduate Program in Biologic Sciences: Physiology, UFRGS (RV); Laboratory of Pain and Neuromodulation at Hospital de Clínicas de Porto Alegre (HCPA) (MDGLT, AD, APB, WC), Porto Alegre; Service of Pain and Palliative Care at HCPA (WC), Brazil; and Harvard Medical School, Department of Physical Medicine and Rehabilitation (FF), Boston, MA.

## Abstract

Based on the hypothesis that an imbalance in excitatory and inhibitory input is a central mechanism of knee osteoarthritis chronic pain (KOACP), this exploratory study had the following aims: to compare whether the function of the descending inhibitory pain pathway is associated with the state of inhibition in the corticospinal system indexed by the motor-evoked potential (MEP) and the cortical salient period (CSP) in patients with severe osteoarthritis (OA) and healthy controls; and to determine if there is correlation between the measures of intracortical inhibition (CSP, MEP) with changes on the numerical pain scale (NPS [0–10]) in KOACP during a conditioned pain modulation (CPM)-task considering the effect of self-reported function assessed by the Western Ontario and McMaster Universities Osteoarthritis Index (WOMAC) and analgesic use.

In a cross-sectional study, we included females (n = 21), with disability by pain or stiffness due to KOACP and healthy controls (n = 10), aged 19 to 75 years. The motor cortex excitability parameters (MEP and CSP) were assessed using the transcranial magnetic stimulation. We assessed the pain and disability by the WOMAC, and change on NPS (0–10) during CPM-task.

A Multivariate analysis of covariance revealed that the adjusted mean (SD) on the MEP amplitude was 13.53% higher in the OA than in healthy subjects (1.33 [0.49] vs 1.15 [0.13]), respectively (*P* = 0.16). The adjusted mean (SD) on the CSP observed in OA patients was 23.43% lower than in healthy subjects (54.54 [16.10] vs 70.94 [22.87]), respectively (*P* = 0.01). The function of the descending pain modulatory system assessed by change on NPS (0–10) during a CPM-task was negatively correlated with the cortical excitability parameter indexed by the CSP (*P* = 0.001). Also, the CSP was negatively correlated with the pain and disability assessed by the WOMAC index.

These findings support the hypothesis that the change in cortical plasticity in KOACP is associated with less intracortical inhibition, as measured by the CSP. These results show that the neural change in the motor cortex in KOACP is associated with pain and disability levels, and also with decreased activation of the endogenous pain-modulating system by a CPM-task.

## INTRODUCTION

Osteoarthritis (OA) is the most important cause of pain and limitation in the older population.^1^ It is associated with chronic inflammation in somatic structures, which alters the afferent sensory inputs and leads to plastic changes in the nervous system.^2^ OA might lead to segmental sensitization^3^ and promote central sensitization (CS),^4^ a phenomenon that comprises expansion of the receptive field, a lower pain threshold, hyperalgesia inside and outside of sensitized areas,^5,6^ and the presence of widespread pain.^6^ Total knee replacement (TKR) is indicated in end-stage OA to reduce pain and disability.^7^

Although TKR surgery may produce a complete resolution of pain in a large percentage of patients (up to 73%) within the first 2 to 7 years after surgery,^7^ persistent pain associated with physical disability has been reported in approximately 15% to 20% of patients.^8^ Whereas particular variables have been consistently associated with poor pain outcomes, such as pain catastrophizing and preoperative pain,^9^ the dysfunction of endogenous pain modulatory systems also provide insight to identifying patients prone to developing increased postsurgical pain^10^ and postoperative chronic pain. Convincing evidence exists to support that the descending modulatory systems in chronic pain are disrupted; shifting from a state of inhibition to a maladaptive state of facilitation.^11^ Also, functional magnetic resonance imaging (fMRI) studies have demonstrated that compared with healthy subjects, patients with OA demonstrate an increased vigilance and a decreased ability to disengage from pain.^12^ These changes were associated with abnormal activity in the cingulate cortex, the amygdala, the insula, the nucleus accumbens (NAc), and the prefrontal areas.^11^

In fact, long-term pain induces cortical reorganization involving the primary motor cortex (M1), which has been a target to assess the cortical excitability and to treat chronic pain conditions. Several studies have shown that M1 stimulation improved pain management outcomes in patients with chronic pain, such as patients with fibromyalgia,^13,14^ trigeminal neuralgia,^15^ phantom pain,^16^ chronic migraine,^17^ low back pain^18^ and myofascial pain syndrome.^19^ Also, the M1 is a target that allows us to characterize pathophysiological consequences associated with chronic pain at the motor cortex by neurophysiological measurements made by transcranial magnetic stimulation (TMS).^20^ Among these parameters, the increase in the motor-evoked potential (MEP) is considered a basic index of corticospinal excitability^21^ and its amplitude has been observed after painful experiences^22^ and under experimental pain.^23^ Also, it has been reported that neuropathic pain leads to a disinhibited state indicated by a shortened cortical silent period (CSP).^24^ TMS protocols using paired pulse suggests that the inhibition in the motor cortex assessed by CSP tap into different mechanisms. Gamma aminobutyric acid (GABA)-B agonists, such as baclofen, was shown to enhance the CSP.^25^ Indeed, the early part of the CSP relies on spinal inhibition.^26^

Considering that the chronic pain associated with OA reduces quality of life and given the expected exponential increase in the number of primary TKA, a better comprehension of the relationship between the intracortical inhibition and the potency of the descending inhibition system could improve future therapeutic approaches in OA. Thus, based on the hypothesis that an imbalance in excitatory and inhibitory inputs plays a role in the central mechanism of knee osteoarthritis chronic pain (KOACP), this exploratory study had the following aims: to compare whether, in patients with severe OA and healthy controls, the function of the descending inhibitory pain pathway is associated with the state of inhibition in the corticospinal system assessed by both the MEP amplitude and the CSP; and to determine if there is correlation between the measures of intracortical inhibition (CSP, MEP) with changes on the Numerical Pain Scale (NPS [0–10]) in KOACP during a conditioned pain modulation (CPM)-task considering the effect of self-reported function assessed by the Western Ontario and McMaster Universities Osteoarthritis Index (WOMAC) and analgesic use.

## METHODS

### Methods

The “Methods” and “Results” sections are reported according to the Strengthening the Reporting of Observational Studies in Epidemiology (STROBE) guidelines.^27^ The Ethics Committee at the Hospital de Clínicas de Porto Alegre (HCPA) approved the study (Protocol No. 11–0013). According to the Declaration of Helsinki, all patients provided written informed consent to participate.

#### Design Overview, Setting, and Participants

A cross-sectional study was performed at HCPA, Brazil, between March 2014 and December 2014. Patients were recruited from the general population through public postings in different healthcare units and referrals from physicians in the Psychiatry and Chronic Pain Service at HCPA. Eligibility criteria were designed to study a group of patients who were potentially appropriate candidates for unilateral knee arthroplasty. The sample comprised of 21 right-handed women meeting inclusion criteria of being aged 50 years or older^4^ and experiencing moderate or intense pain or stiffness in the knee. Also, they needed to present functional impairments for at least 6 months that were not controlled with medical therapy.^28^ The baseline interview included the WOMAC, a validated instrument to assess pain, stiffness, and functional limitations related to OA.^29^ To be eligible, they could report “moderate,” “severe,” or “extreme” pain or stiffness in response to at least 1 of the 5 pain questions (pain with walking, climbing stairs, reclining, sitting, or standing). They needed to report a positive answer for 2 stiffness questions (morning stiffness, stiffness later in day), and also reporting whether they experience “moderate,” “severe,” or “extreme” difficulty with at least 1 of the 17 activities. Additionally, the radiographs of all knees were evaluated for the degree of OA by 1 physiatrist with over 10 years of experience in OA rehabilitation. This was conducted using the Kellgren–Lawrence (K-L) grading scale of 3 to 4,^30^ because it proved to be highly reproducible to grading severity of knee OA.^30^ The exclusion criteria were as follows: accompanied orthopedic, rheumatic, or neurological pathologies; surgery on the affected areas in the prior 6 months; habitual use of corticosteroids or other uncompensated chronic pathologies. Additionally, patients were excluded if they had a body mass index (BMI) of >35 m/kg^2^ or if they had contraindications to TMS.^31^

**FIGURE 1:**
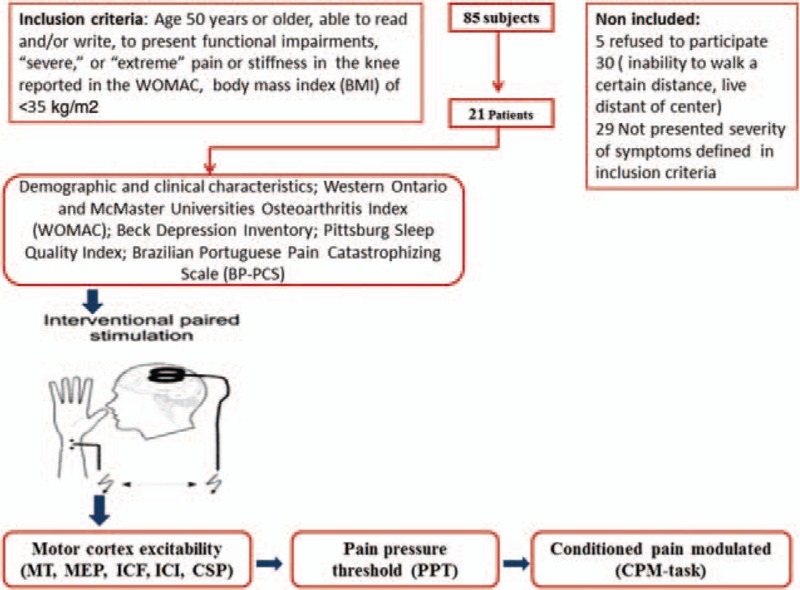
The sequence of assessments. Healthy controls underwent the same sequence of assessments, except the questionnaires regarding, sleep, pain, and depression.

Healthy right-handed controls were recruited from the general population using public postings. They were asked to complete screening questionnaires, and were excluded if they were experiencing any painful condition (either acute or chronic); used analgesics or corticosteroids; had any rheumatologic, psychiatric, or neurological disorder; had abused alcohol or psychotropic substances during the 6 months previous to the screening; or if they were using medications with known effects on the central nervous system (CNS). In addition, they were excluded if presented contraindications to TMS.^31^ The sequence of assessments is presented in Figure 1.

#### Dependent Variables

The dependent variables of interest were measurements of the intracortical inhibition indexed by CSP and MEP.

The left primary motor cortex (M1) parameters were assessed using the TMS MagProX100 stimulator (MagVenture Company, Lucernemarken, Denmark) through a figure-eight coil (MagVenture Company). It was assessed before the pain pressure threshold assessment. Ag-AgCl electrodes were placed over the first dorsal interosseous (FDI) belly muscle and in its corresponding tendon on the distal phalanx of the index finger. The responses to stimuli were recorded from the FDI muscle of the right hand by surface electromyography (EMG).

Each patient was seated in a comfortable chair and informed about the TMS procedure, including possible sensations that might be experienced. The amplitudes of the single and paired pulse TMS, and the latency and the measures of the CSP during the experiment were recorded on an Excel spreadsheet. The data were analyzed offline on a personal computer.

To identify the motor “hot spot,” the coil was placed over the left M1 tangentially to the scalp at a 45° angle to the sagittal line. The motor threshold (MT) was defined using the lowest stimulus to induce 50% of the evoked potentials of the resting FDI.^32^ To ensure constant placement of the coil throughout the TMS assessments, the site was marked with a soft-tipped pen. Firstly, the MT was determined using the lowest stimulus to elicit evoked potentials in the resting FDI, with a minimum amplitude of 50 μV peak-to-peak, in at least 5 of 10 (at least 50%) of successive trials).^32^

Single-pulse measures including the MEP and the CSP were recorded at an intensity of 130% of the MT. The MEP value was the elicited evoked potential with a 1-mV peak-to-peak amplitude. The mean of 10 consecutive trials were recorded. For the CSP, patients were instructed to perform isometric voluntary contractions with approximately 10% of maximal contraction of the FDI. The transient silence during the isometric voluntary EMG activity was elicited in the tonically contracting FDI muscle at approximately 10% of the maximal voluntary contraction, and the CSP was preceded by the MEP.^33^ Ten consecutive trials were recorded. The paired-pulse measurements included the short-interval intracortical inhibition (SICI) with interstimulus intervals of 2 milliseconds and the intracortical facilitation (ICF) with interstimulus intervals of 12 milliseconds.^34^ To define the individual MT, the first subthreshold stimulus was set at 80%, whereas the supra-threshold stimulus was set at 130% of the MT. The intensity of the supra-threshold test stimuli was adjusted to elicit the test stimuli with peak-to-peak amplitude of approximately 1 mV. At the level of the primary motor cortex, the reduction of the test MEP elicited by TMS is considered to reflect inhibition^34^ and the increase of the test MEP elicited by TMS is considered to reflect facilitation at the level of the primary motor cortex.^21^ Thirty recordings (10 for each ICI, ICF, and the test stimulus) were produced in a random order with an interval of approximately 8 seconds between each pulse. The paired-pulse measurements were analyzed by calculating their individual index (mean ICI/mean of the test stimulus; mean ICF/mean of the test stimulus).^35^ These parameters were assessed before and after 2 minutes of rest.^36^

#### Independent Variables

All psychological tests used in this study were validated for the Brazilian population.^37,38^ The patients’ depressive symptoms were assessed using the Beck Depression Inventory,^39^ and sleep quality was assessed using the Pittsburgh Sleep Quality Index.^40^ Pain catastrophizing was assessed using the Brazilian Portuguese Pain Catastrophizing Scale (BP-PCS).^41^ A standardized questionnaire was used to assess comorbidities and demographic data. The WOMAC was used to evaluate self-report of knee-specific impairment based on symptoms during the preceding 48 hours. The WOMAC assesses the pain, joint stiffness, and perceived disability associated with OA to determine the overall impact on a patient's perceived function. It comprises 24 questions with responses given to each using a Likert scale. The pain subscale consists of 5 possible responses: no (0), mild (1), moderate (2), severe (3), or extreme (4) problems. This 20-point scale (range of 0–20) assesses pain in everyday situations (eg, walking on flat surfaces). The stiffness subscale is a 2-item (range 0–8), 10-point scale that assesses perceived knee stiffness after walking and at the end of the day. The disability subscale is a 17-item assessment of perceived physical function in a variety of everyday situations (eg, getting into and out of a car). The WOMAC is a valid, reliable, and responsive instrument that is commonly used to assess pain and disability in studies of knee OA.^29^ A total WOMAC score (range 0–96) is calculated by summing the items for all 3 subscales.^42^

To measure the pressure pain threshold (PPT), we asked patients to differentiate the perception of pressure versus the perception of “onset of pain.” The patient was instructed to report the perception of pain onset verbally. A trained investigator assessed the pain threshold. An experienced rehabilitation physician (MGT) systematically evaluated superficial and deep hyperalgesia by assessing the PPT using an electronic algometer (J Tech Medical Industries). Three successive readings taken at intervals of 3 to 5 minutes were used to define the PPT in kgf/cm^2^ (lb/cm^2^). The PPT was recorded at the site of greatest sensitivity where the device had a 1-cm^2^ hard-rubber probe applied to the myotome and sclerotome structures at the L1-L5 and S1-S2 dermatomes at the knee with greater pain (knee hyperalgesia).

To test the CPM, we used the Tousignant-Laflamme et al^43^ protocol, and the experimental pain stimulus used was in accordance to the guidelines for the cold-pressor task (CPM-task).^44^ The CPM-task is a strong nociceptive stimulus applied over a large body surface area^44^ that takes place over a lengthy time span.^45^ The CPM-task allows us to modify the descendent pain modulatory system.

To assess the CPM-task, the patient immersed the nondominant hand in cold water (0–1°C) for 1 minute. During the last 30 seconds of the cold-water immersion, the PPT procedure was administered to the dominant forearm. During the entire experiment, the cold-water temperature was maintained constant. The PPT that elicited pain ratings of 6/10 on the NPS (0–10) (PPT60) was used for the first PPT before the CPM-task (PPT0). After a short break, the PPT0 was applied at the S1-S2 dermatome at the knee of the leg with higher hyperalgesia. After PPT0, the CPM-task was used to trigger the CPM. One minute after the CPM-task, we applied the second PPT (PPT1). To quantify the CPM, the mean pain rating of PPT1 was subtracted from the first PPT0 before the CPM-task (PPT1); negative values indicate inhibitory CPM.

Analgesic use was defined by an average of analgesics used per week during the previous month. For data analysis, analgesic use was included as a dichotomous variable (the use of analgesics less than 4 days per week or the use on more than 4 days per week). This approach was chosen because patients with chronic pain rescue analgesic use changes each week, depending on their level of pain.

#### Efforts to Address Potential Sources of Bias

To reduce assessment bias, only 1 researcher (MGT) was involved in all of the assessments. The evaluator (MGT) is a practicing physiatrist of the outpatient clinic at HCPA with vast clinical expertise, who is well trained to make the TMS measures. Also, the evaluator was trained to apply clinical scales and PPT assessment, and also in the care of chronic pain patients. In our study, all patients were submitted to a clinical evaluation by the same physician (MGT), who had many years of experience in treating patients with OA, to revise the severity of OA and inclusion criteria. The algometer used to make measurements was manufactured by (J Tech Medical Industries).

#### Sample Size

The number of patients was estimated based on a type I and type II error of 0.05 and 0.20, respectively, and in anticipation of an effect size (*f*_2_ = determination coefficient) of 0.4 for the multiple hierarchical regression analysis allowing for 2 predictors (the post-hoc Statistical Power Calculator for Hierarchical Multiple Regression: http://www.danielsoper.com/statcalc3/calc.aspx?id).^46^ A sample of 18 patients was chosen to account for unexpected factors that would decrease the study power such as increased variability of the sample or missing data. A sample of 21 patients would detect an effect size for correlations of 0.4, with a power of 88% at a 0.05 alpha level.

### Statistical Analysis

Descriptive statistics were used to summarize the main characteristics of the sample. To evaluate if continuous variables presented criteria to normal distribution, we used skewness/kurtosis tests. To compare continuous variables, we used the *t* test for independent samples and the chi-square or Ficher exact test for categorical variables.

A Multivariate analysis of covariance (MANCOVA) was used to assess the relationship between the dependent variables, the cortical excitability parameters (MEP, CSP) with the main interest independent variable, and the change on the NPS (0–10) during the CPM-task in patients with OA and healthy subjects. The covariate included in the model was age. We used Bonferroni multiple comparison test to adjust the differences for multiple comparisons.

A regression multiple analysis was used to explore the relationship between the change on NPS (0–10) during the CPM-task and cortical excitability parameters (CPS and MEP) in OA patients. This procedure was done to adjust this analysis for potential confounding factors in OA patients, such as analgesic use and disability assessed by the WOMAC index. Also, a regression analysis was used to generate the scatter plot of correlation between CSP and change on NPS (0–10) during the CPM-task. The data were analyzed using SPSS version 22.0 (SPSS, Chicago, IL).

## RESULTS

### Baseline Characteristics

Twenty-one women with OA participated in this study along with 10 healthy women. The baseline demographics, psychological characteristics, and cortical excitability parameters are shown in Table 1. A statistical significant difference was observed between OA patients and healthy subjects in age, years of formal education, and reduction on NPS (0–10) during the CPM-task and resting MT.

**TABLE 1:**
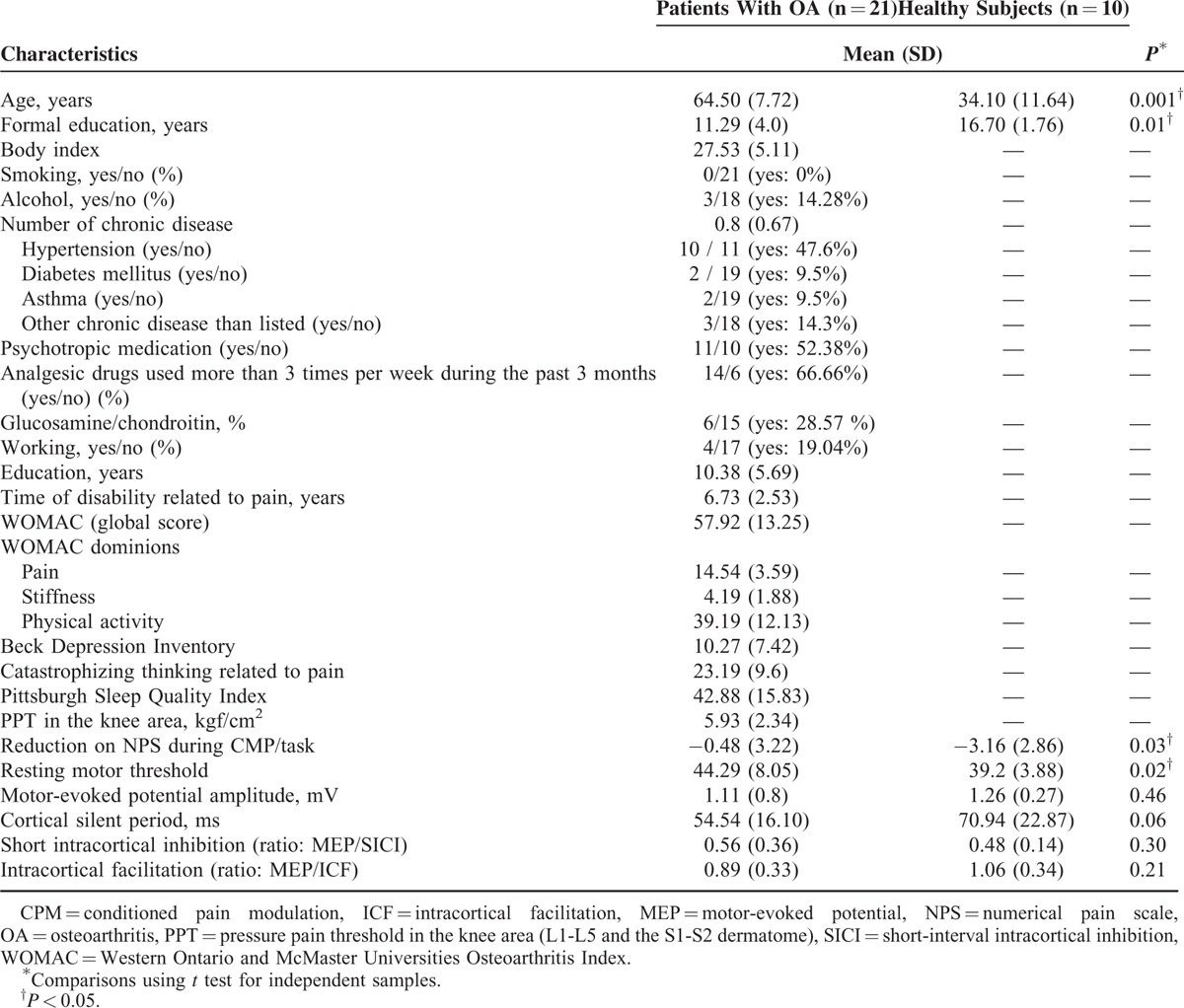
Clinical and Demographic Characteristics, Psychological State, and Measures of Intracortical Inhibition of the Sample (n = 31)

### Relationship Between Cortical Excitability Parameters and Descending Pain Modulatory System in Patients with OA and Healthy Subjects

**FIGURE 2:**
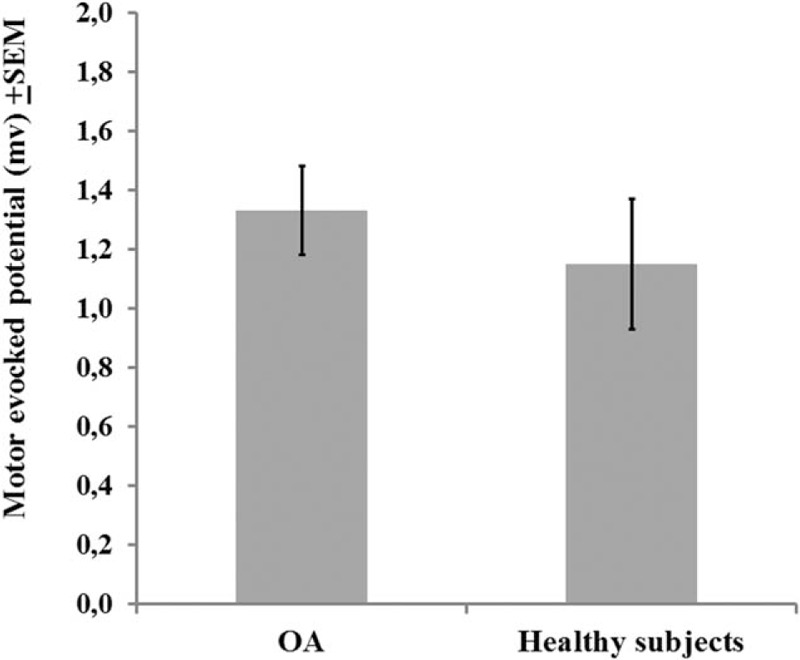
Comparisons between groups osteoarthritis (OA) or healthy subjects, on motor-evoked potential (MEP) (n = 31). The error bars indicate standard error of mean (SEM). The bars indicate means of each groups (OA or healthy subjects) compared by Multivariate analysis of covariance (MANCOVA).

A MANCOVA was used to adjust, by age, the relationship between the groups (OA patients or healthy subjects) and their outcomes related to cortical excitability measurements (CSP and MEP amplitude) with the descending pain modulatory system as assessed by the reduction on NPS (0–10) during CPM-task (Wilks *λ* = 0.43, F [4] = 5.08, *P* < 0.01). The power of this analysis was 0.92%. The adjusted determination coefficient of this model is *R*^2^ = 0.47 (ie, the variables included in the model explain 47% of the variance in the outcomes variables). This analysis revealed that the function of the descending pain modulatory system, assessed by change on NPS (0–10) during CPM-task, is negatively correlated with the cortical excitability parameter indexed by the CSP (*P* < 0.05) (Table 2). This analysis revealed that the MEP amplitude was not statistically different between OA patients and healthy subjects (*P* > 0.05). The adjusted mean (SD) on the MEP amplitude observed in OA was 13.53% higher than in healthy subjects (1.33 [0.49] vs 1.15 [0.13]), respectively (Figure 2).

**TABLE 2:**
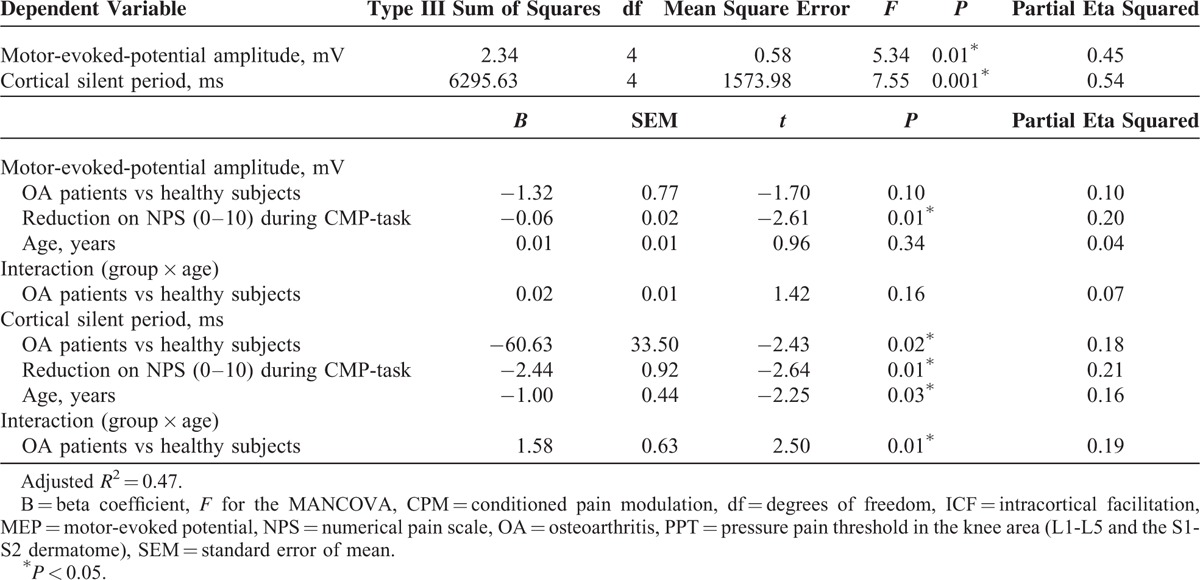
Multivariate Analysis of the Relationship Between Measures of Intracortical Excitability and Change on NPS (0–10) During CPM-Task in Patients with OA and Healthy Subjects (n = 31)

**FIGURE 3:**
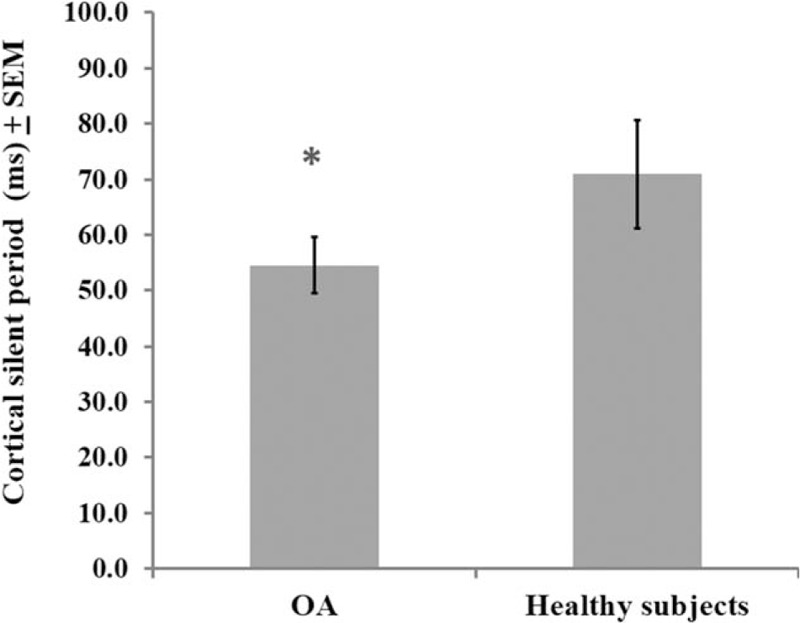
Comparisons between groups osteoarthritis (OA) or healthy subjects, on cortical silent period (CSP) (n = 31). The error bars indicate standard error of mean (SEM). (∗) Asterisks positioned above the bars indicate differences between groups (OA or healthy subjects) assessed by Multivariate analysis of covariance (MANCOVA) with post hoc Bonferroni multiple comparison test.

Also, the MANCOVA analysis (Table 2) shows that age was negatively correlated to the CSP. Accordingly, healthy subjects presented a longer CSP. The adjusted mean (SD) on the CSP observed in OA patients was 23.43% lower than in healthy subjects (54.54 [16.10] vs 70.94 [22.87]), respectively (Figure 3). We observed that in healthy subjects, the age was negatively correlated to the CSP, whereas in OA patients, the direction of this relationship was inverse. Thus, even having older OA patients in comparison to healthy subjects, the older age was not enough to prolong the CSP as would be done in healthy subjects of the same age, and in turn, OA patients presented shorter CSP (Table 2). Thus, this suggests that the inhibition within OA patients due to the neuroplastic changes was induced by chronic pain.

The MANCOVA analysis (Table 2) showed that the MEP amplitude and the CSP were negatively correlated with change on the score of the NPS (0–10) during the CPM-task. That is, the increase in the CSP was correlated with a higher change on the NPS (0–10) during the CPM-task, or vice versa (Table 2). It is important to remember that a higher change on the NPS (0–10) during the CPM-task indicates that the heterotopic stimulus was more effective; thus, the difference on NPS (0–10) (PPT1 minus PPT0) produced a higher negative value. Thus, this explains the coherence this negative correlation.

Also, the increase of the MEP amplitude was negatively correlated with the change on the NPS (0–10) during the CPM-task (Table 2). Considering that higher MEP amplitude indicates higher excitability on the cortical–spinal pathway, it should be plausible to expect a positive correlation, because it would be less prone to modulating the nociceptive stimulus. However, the MEP amplitude was not statistically different between groups (OA and healthy subjects) (Table 2).

**FIGURE 4:**
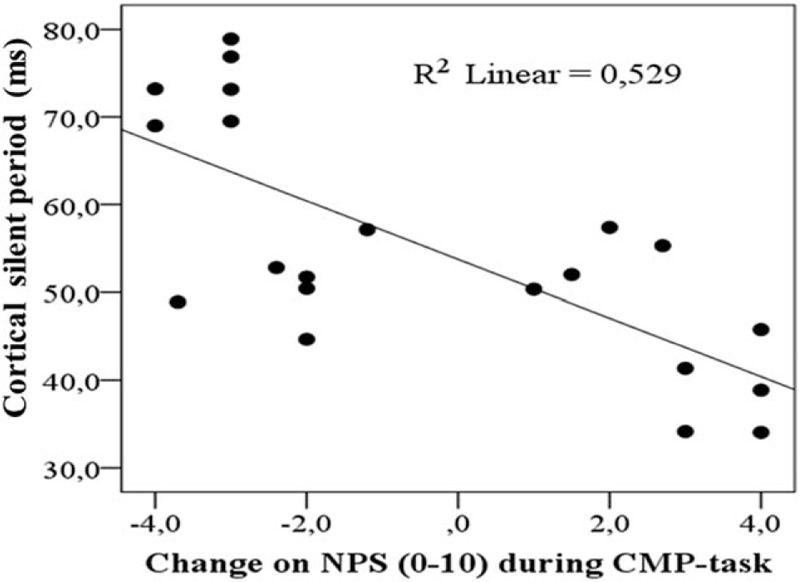
Scatter plot of the correlation between changes on NPS (0–10) during CPM-task and the current silent period (CSP) in patients with osteoarthritis (OA) (n = 21). CPM = conditioned pain modulation, NPS = numerical pain scale.

An important question is to identify if this result could be explained by other confounding factors. Thus, we run a multiple regression analysis only with the OA patients (Table 3). In this model, the relationship between the change on the NPS (0–10) during the CPM-task and cortical excitability parameters (CPS and MEP) was adjusted by analgesic use and the self-reporting of pain and disability assessed by the WOMAC index. The multiple regression analysis confirmed an inverse correlation between the CSP with the change on the NPS (0–10) during the CPM-task, but not with the MEP amplitude (Table 3). The scatter plot of the raw CSP and change on the NPS (0–10) during the CPM-task is presented for illustrative purposes in Figure 4. The Pearson correlation coefficient (*r*) was −0.72 (95% confidence interval [CI] −0.87 to −0.38) and the coefficient of determination (*R*^2^), that is the proportion of the variance explained by the association between the change on NPS (0–10) during CPM-task and the CSP was 52% (*R*^2^ = 0.52).

**TABLE 3:**
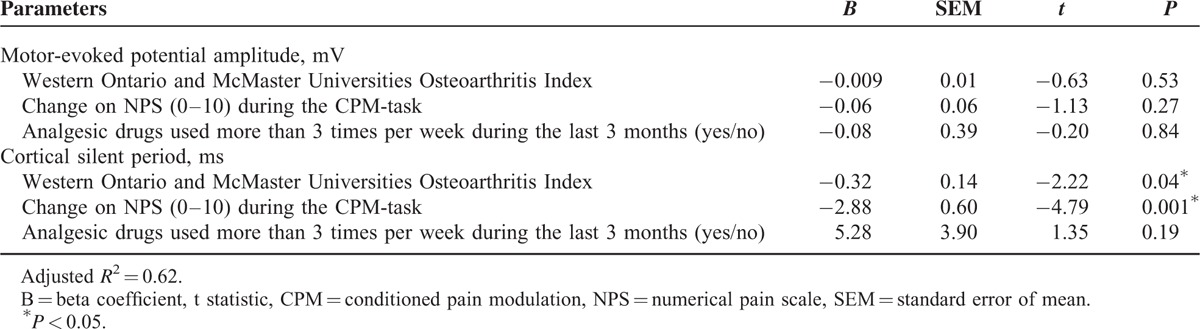
Linear Regression of the Relationship Between Measures of Intracortical Excitability and the Function of Descending Pain Modulation Adjusting by Potential Confounding Factors (n = 21)

## DISCUSSION

This study extends the literature about the correlation between measures of the intracortical disinhibition as indexed by CSP and the dysfunction in the descending control of nociceptive processing as indexed by lower activation of the CPM-task in KOACP. Whereas, a lower CSP indicates that the trigger to induce a membrane potential is lower, thereby, there is less inhibition. A higher value of the change on the NPS (0–10) during the CPM-task indicates that the heterotopic stimulus to induce CPM was less effective. This assignment on descending pain modulatory system may explain the inverse correlation between the CSP with the disability related to pain as assessed by the total WOMAC.

Our finding suggests that sustained peripheral inflammation increases the excitability of ascending nociceptive pathways, which induces dysfunction in descending modulatory systems, as assessed by the change on the NPS (0–10) during the CPM-task. At the cortical level, a shorter CSP reflects the decreased excitability of the inhibitory interneurons. This disinhibited state observed in OA, but not in healthy subjects, support the hypothesis that the chronic pain in OA induces a disinhibitory effect at the intracortical level, and also in the descending pain modulatory system. Whereas, the intracortical disinhibition is in agreement with what has previously been described in neuropathic and fibromyalgia pain.^47^ The intracortical disinhibition state observed in this study is in disagreement with findings of a previous report, which did not find any significant changes in the motor cortical excitability in OA patients compared with healthy subjects.^48^ It is possible that the severity of OA and the long-term pain level could explain this divergence. Our sample patients were shown to have moderate to severe knee OA, intense chronic pain, and disability during more than 5 years, whereas the above mentioned study noted OA pain in the hands, which carries a mild severity.^49^

In the present study, higher disability was inversely correlated with the CSP. Given that the CSP index assesses the function of GABAergic transmission,^50^ it is conceivable that sustained pain—in this case, triggered by peripheral inflammation—could lead to a cascade of events resulting in dysfunctional inhibitory function. Part of this defective cortical inhibition could be explained by recent evidence showing decreased gray matter volume associated with chronic pain syndromes, including OA.^51^ Central reorganization in chronic pain seems to lead to an inhibitory state that might facilitate activation in nonpain networks, such as the primary motor cortex. Thus, disturbances in the GABAergic and glutamatergic intracortical networks might explain the disinhibition found in KOACP.

The shorter CSP suggests a decrease of GABAergic neurons because they are responsible to exert rapid synaptic inhibition via GABA-B receptors.^52^ This activation of the GABAergic system governs the state of inhibitory interneurons within M1.^52^ Also, it is known that the balance between inhibitory and excitatory systems is influenced by age. Older subjects present a slower motor response and a decline in the modulation of the corticospinal activity system.^53^ Whereas this association is consistent in healthy subjects,^54^ our results suggest that chronic pain leads to changes in favor of excitability, as demonstrated by a shorter CSP. Also, the adjusted analysis shows that age is intrinsically associated with slower intracortical excitability, because the direction of correlation between the CSP and age changed when we analyzed the interaction of age with the group (OA or healthy subjects) (Table 3). However, the independent association between the CPM-task and the CSP persisted even after adjustment by age (Table 2). Thereby, it is unlikely that controls’ age would modify the present findings because the relationship between the CPM-task and CSP persisted even when we included only patients with OA (Table 3). However, we cannot assume that the effect of age as a confounding factor was entirely controlled. Although an ideal strategy to validate our results is to compare them with healthy subjects, in a real-life scenario, it is complex to find controls to match the profile of our sample of OA.

Thus, changes in cortical plasticity in OA could be explained by a steady pain induced by a peripheral neural lesion, which increases the synaptic efficacy of neural structures involved in pain processing likewise occurring on a neuropathic lesion. The CSP in this study suggests that ongoing nociception from knee-related structures is essential to the chronic nature of this process and the development of sensitization.^55^ The association between the severity of pain and the disinhibition at cortical and infra-cortical levels highlights that knee hyperalgesia is an important generator of pain and sensitization as previously demonstrated in knee arthroplasty.^4^ However, cumulative evidence suggests that inflammation leads to increased hyperalgesia, which concurs with a lack of descending inhibitory pain mechanism.^9^ This finding was demonstrated in the present study and it is also supported by previous reports.^9^ It has been demonstrated that this diminished activity of the descending inhibitory interneuron activity is a consequence of a decreased synthesis of neurotransmitters (GABA and glycine), a diminished activity of serotonin and norepinephrine.^56^

In this study, the lower activation of the CPM induced by the CPM-task was correlated negatively with the CSP (Figure 4), which suggests that the lower activation of the CPM by the CPM-task is related to higher intracortical disinhibition. Based on a cross-sectional analysis, this finding indicates that worsening of the descending pain inhibitory system function is associated with a loss of cortical pain inhibition. This is likely a consequence of higher pain intensities and longer durations, which induce more facilitated temporal summation compared with lower pain intensities and shorter durations of pain.^57^

The downward negative spiral of pain and central disinhibition has severe clinical consequences, which are as follows: it increases pain and local knee hyperalgesia and it is associated with a loss of cortical inhibitory mechanisms. Although the design of this study prevents determining the deterioration in the central pain modulatory system, it does permit us to better understand the dysfunctional process of disinhibition at cortical and intracortical regions in severe KOACP. Thus, the pieces of evidence these findings possess may hold important clinical implications such as to support an understanding of the bidirectional pathways between peripheral inflammation and central brain changes in OA; to select the best therapeutic approach based on the neurophysiological phase state of each patient, because chronic pain has been associated with unfavorable pain outcomes after knee arthroplasty^58^; to determine strategies to manage patients with a higher risk of more severe chronic pain after knee arthroplasty, which includes anesthetic and analgesic approaches.^4^ Also, it improves the understanding of underlying neurophysiological mechanisms of chronic pain in OA, which could give support to plan new neuromodulatory approaches to induce a top down (ie, direct current stimulation [tDCS]) and bottom-up modulation technique (ie, dry-needling) or pharmacological interventions.

The small sample size is a limitation of this study. The study design is a limitation because it is not possible to determine a causative effect. The use of TMS assesses the neurotransmitter system activity in an indirect manner, and it has been shown to have relatively low specificity. However, TMS is a useful tool for neurophysiological assessment because it induces activity and evaluates the response of the subject. In this study, only females were evaluated, taking into account that sex differences in pain perception and modulation are a controversial topic.^59^ This limitation restricts the possibility of a direct comparison with other studies, but has the advantage of avoiding possible contamination of the data. Because OA is more prevalent in females,^60^ these results might have greater clinical implications. Although we recruited healthy volunteers to assess the relationship between the cortical inhibitory function and the descendent pain inhibitory system, it is worth noting that our control sample was younger on average.

These findings support the hypothesis that a change in cortical plasticity in KOACP is associated with less intracortical inhibition, as measured by the CSP. These results show that this neural change in the motor cortex in KOACP is associated with pain and disability levels, and also with decreased activation of the endogenous pain modulating system by the CPM-task.
